# PRC1 uncomplexed

**DOI:** 10.1016/j.stemcr.2022.04.010

**Published:** 2022-05-10

**Authors:** Sanne Schouten, Nick Bovee, Zicong Liu, Hendrik Marks

**Affiliations:** 1 Department of Molecular Biology, Faculty of Science, Radboud University, Radboud Institute for Molecular Life Sciences (RIMLS), 6525GA Nijmegen, the Netherlands

## Abstract

Epigenetic enzymes are critically involved in gene regulation during lineage commitment. In this issue of *Stem Cell Reports*, Zhu et al. (2022) unravel extensive redundancy between subunits of the epigenetic regulatory Polycomb Repressive Complex 1 using a systematic knockout strategy in mouse embryonic stem cells.

## Main text

The generation of an organism requires a large range of specialized cell types to arise from a single fertilized oocyte. This intriguing process is tightly regulated during early development and onwards. The diversification of the pluripotent epiblast into ectoderm, endoderm, and mesoderm demands cell fate commitment to happen at the right place, at the right time. From a gene regulatory point of view, this process involves transcription factors that act in concert with chromatin modifiers to coordinate gene expression. One of the key chromatin modifiers during development, and arguably the best studied, is represented by the Polycomb Group (PcG) proteins. These proteins were first described in *Drosophila melanogaster* and were found to be highly conserved among species (Schuettengruber et al., 2017). The PcGs are involved in the establishment of poised chromatin, preparing genes encoding developmental regulators for activation. The importance of PcGs in this process is illustrated by dysregulation of PcGs being associated with developmental disorders and cancer (Piunti and Shilatifard, 2021).

In mammals, several PcG proteins assemble into two types of polycomb repressive complexes: Polycomb Repressive Complex 1 (PRC1) ubiquitinates histone H2A at lysine 119 (H2AK119ub1), while PRC2 deposits one or more methyl groups at lysine 27 of histone H3 (H3K27me1/-2/-3) (Schuettengruber et al., 2017). The PRC-catalyzed H2AK119ub1 and H3K27me3 are considered repressive chromatin marks that maintain gene silencing. PRC1 is further divided into canonical PRC1 (cPRC1) and non-canonical PRC1 (ncPRC1). cPRC1 contains a CBX subunit that is recruited to PRC2-deposited H3K27me3, whereas ncPRC1 lacks a CBX subunit and functions through an elusive PRC2-independent recruitment mechanism (Schuettengruber et al., 2017). The functional interplay of cPRC1, ncPRC1, and their subunits during early development have thus far remained largely enigmatic. In this issue of *Stem Cell Reports*, Zhu et al. (2022), show a clear functional distinction between cPRC1 and ncPRC1 in maintaining lineage commitment potential in mouse embryonic stem cells (mESCs). Furthermore, they show substantial redundancies between the subunits of ncPRC1.

**Figure 1:**
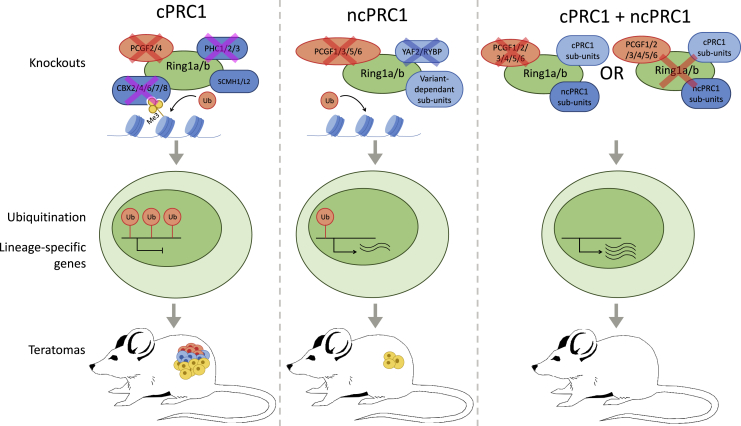
The role of cPRC1 and ncPRC1 in pluripotency Systematic KO of cPRC1 subunits in mESCs does not affect chromatin ubiquitination levels, expression of lineage-specific genes, nor the capacity of mESCs to form teratomas. In contrast, ablation of ncPRC1 results in greatly reduced ubiquitination levels and loss of pluripotency, in particular if combined with KO of cPRC1. Red cross: KO following pluripotency marker assay, teratoma assay, and RNA-seq analyses; pink cross: KO following pluripotency marker assay and teratoma assay; blue cross: KO following pluripotency marker assay. Differently colored cells in teratoma represent the three germ layers, with yellow being endoderm.

The cPRC1 and ncPRC1 complexes both have a RING1A/-B catalytic core but are otherwise different. In cPRC1, the core assembles with PCGF2/-4, CBX2/-4/-6/-7/-8, PHC1/-2/-3, and SCMH1/-L2. In ncPRC1, the core assembles with PCGF1/-3/-5/-6, RYBP or YAF2, and accessory proteins (Schuettengruber et al., 2017). The vast number of subunit variations has previously challenged research aimed at clarifying the roles and interplay of cPRC1 and ncPRC1. Zhu et al. (2022) have now approached this challenge by applying a powerful, systematic screen of single and combined knockout (KO) mESC lines, using phenotyping, transcriptome profiling, and teratoma assays as readout (Figure 1). These studies show that knocking out individual cPRC1-specific subunits does not affect the pluripotency and self-renewal capacity of mESCs. The KO of all paralogs within one family of cPRC1-associated subunits, for example *Pcgf2* together with *Pcgf4*, did not affect these properties of mESCs either, in line with previous observations that cPRC1 has limited contribution to gene repression (Fursova et al., 2019). In contrast to cPRC1, ncPRC1 appeared essential in maintaining the mESC state. Although a dual KO of *Rybp* and *Yaf2* did not affect pluripotency of mESCs, a combined KO of all ncPRC1-associated *Pcgf* paralogs (*Pcgf1*, *-3*, *-5*, and *-6*) resulted in loss of pluripotency and strongly reduced teratoma formation. Surprisingly, the self-renewal capacity of these multi-*Pcgf*-KO mESCs was retained. When mESCs were ablated of both ncPRC1 and cPRC1 by combined KO of *Pcgf1-6* or *Ring1a/-b*, pluripotency as well as the capacity for self-renewal and teratoma formation were lost. This shows that cPRC1 itself is non-essential for maintaining pluripotency but acts in synergy with ncPRC1 to maintain the mESC state.

Loss of ncPRC1 concurs with a loss of H2AK119ub and subsequent aberrant expression of lineage-specific genes. Interestingly, mESCs with a single KO of *Pcgf1/-3/-5/-6* display unaffected H2AK119ub levels and do not display abnormalities, in line with findings that PCGFs (Zepeda-Martinez et al., 2020) and ncPRC1 variants (Fursova et al., 2019) largely co-occupy polycomb target sites. This points toward extensive redundancy within the ncPRC1 complex, in which the PCGF subunits jointly safeguard pluripotency and self-renewal capacity. Redundancy in PRC1 extends beyond PCGFs, as *Ring1a* and *Ring1b* act redundantly, with single KO mESCs lacking a clear phenotype (Zhu et al., 2022). Moreover, results from Zepeda-Martinez et al. (2020) suggest that PRC2/cPRC1 and ncPRC1 act redundantly to enforce silencing of shared PRC-target genes in mESCs. The redundancy between the PCGFs in ncPRC1 does not imply that individual PCGF subunits only have shared functions. Despite broadly overlapping target sites, individual PCGF subunits also occupy distinct genomic locations that lack other PCGFs to fulfill distinct gene regulatory functions (Gao et al., 2012; Scelfo et al., 2019). For example, PCGF6-containing ncPRC1 plays an important role in preventing abnormal expression of germ-cell-related genes in mESCs (Liu et al., 2020). Also, PCGFs may serve additional roles, such as stabilizing PRC1 complexes. Zhu et al. (2022) hypothesize that PCGF paralogs act redundantly to increase the stability of RING1A/-B. This hypothesis is supported by their observation that a multi KO of *Pcgf1-6* results in reduced RING1A/-B protein levels. In addition, a PRC1-unrelated regulatory function of PCGFs has been proposed (Scelfo et al., 2019). In accordance, RNA-seq analysis shows that a combined *Pcgf1-6* KO in mESCs results in the deregulation of many genes that are not deregulated upon combined KO of *Ring1a/-b* (which results in complete loss of PRC1 activity). Thereby Zhu et al. (2022) present new evidence for a PRC1-unrelated role of the PCGF proteins.

By means of a thorough characterization of a targeted set of KO mESC lines, Zhu et al. (2022) provide an integrated view of PRC1 and its role in pluripotency. The results lead the way to new objectives, such as elucidating the role and possible redundancy of PRC1 complexes beyond early embryogenesis. It remains unknown whether redundancies of ncPRC1 also manifest in later development, for example during lineage decisions toward and within the germ layers and the associated spatial patterning. To answer such questions, the KO mESC lines that were generated in the current study will be highly useful. A convenient *in vitro* assay of embryogenesis would be inducing gastruloid formation using the newly generated KO mESCs. Gastruloids are three-dimensional aggregates of ESCs with an axially organized array of cell types, making them a useful model to simulate *in vivo* embryogenesis (Beccari et al., 2018). Similarly, lineage decisions beyond gastrulation could be studied using a recently developed *ex-utero* mouse embryogenesis model for late organogenesis (Aguilera-Castrejon et al., 2021). Additionally, mouse blastocysts may be complemented *in vivo* with the KO mESCs to evaluate their chimeric contribution within the various tissues of embryos or adult mice. The conditional KO mESC lines as generated for *Ring1b* and *Pcgf6* enable studying the function of these genes at defined developmental timepoints.

With their studies, Zhu et al. (2022) answer pressing questions about the function of PRC1, focusing on the functionality of individual subunits and their gene-regulatory role. Their results highlight that ncPRC1 is essential in early development, with redundancy in its subunits ensuring robust pluripotency and self-renewal capacity. This redundancy in subunits of PRC1 might have evolved to provide robustness and adjustability to the PRC1 regulatory network. This raises the question whether epigenetic modifiers that catalyze different epigenetic marks—such as the MLL, SMARC, and NuRD complexes—have evolved similar features. Altogether, the authors have provided an exemplary systematic study and have paved the way to further uncomplex PRC1.
